# Introduction of Caveolae Structural Proteins into the Protozoan *Toxoplasma* Results in the Formation of Heterologous Caveolae but Not Caveolar Endocytosis

**DOI:** 10.1371/journal.pone.0051773

**Published:** 2012-12-14

**Authors:** Bao Lige, Julia D. Romano, Vera Sampels, Sabrina Sonda, Keith A. Joiner, Isabelle Coppens

**Affiliations:** 1 Department of Molecular Microbiology and Immunology, Johns Hopkins University Bloomberg School of Public Health Baltimore, Maryland, United States of America; 2 Institute of Parasitology, University of Zurich, Zurich, Switzerland; 3 Arizona Health Science Center, University of Arizona College of Medicine, Tucson, Arizona, United States of America; Emory University School of Medicine, United States of America

## Abstract

Present on the plasma membrane of most metazoans, caveolae are specialized microdomains implicated in several endocytic and trafficking mechanisms. Caveolins and the more recently discovered cavins are the major protein components of caveolae. Previous studies reported that caveolar invaginations can be induced *de novo* on the surface of caveolae-negative mammalian cells upon heterologous expression of caveolin-1. However, it remains undocumented whether other components in the transfected cells participate in caveolae formation. To address this issue, we have exploited the protozoan *Toxoplasma* as a heterologous expression system to provide insights into the minimal requirements for caveogenesis and caveolar endocytosis. Upon expression of caveolin-1, *Toxoplasma* accumulates prototypical exocytic caveolae ‘precursors’ in the cytoplasm. *Toxoplasma* expressing caveolin-1 alone, or in conjunction with cavin-1, neither develops surface-located caveolae nor internalizes caveolar ligands. These data suggest that the formation of functional caveolae at the plasma membrane in *Toxoplasma* and, by inference in all non-mammalian cells, requires effectors other than caveolin-1 and cavin-1. Interestingly, *Toxoplasma* co-expressing caveolin-1 and cavin-1 displays an impressive spiraled network of membranes containing the two proteins, in the cytoplasm. This suggests a synergistic activity of caveolin-1 and cavin-1 in the morphogenesis and remodeling of membranes, as illustrated for *Toxoplasma*.

## Introduction

Cellular uptake of substances from the environment depends on specialized subdomains of the plasma membrane [1]. Among them, caveolae are containers that sequester and transport multiple signaling molecules to different subcellular (non-lysosomal) locations such as the Golgi apparatus [2]–[5]. Caveolae are specialized forms of lipid rafts enriched in cholesterol and sphingolipids [6]. Structurally, they contain an integral membrane scaffold composed of caveolin proteins and a peripheral protein layer made of cavin complexes [7]. These proteins confer to caveolae their characteristic morphology: flask-shaped 60–90 nm plasmalemmal invaginations, as is observed in electron micrographs.

The caveolin family members include caveolin-1 (referred here as CAV1) (α and β), caveolin-2 (α and β) and caveolin-3 [8]. Although similar in structure and function, caveolin proteins differ in their distribution in cells: CAV1 and caveolin-2 form stable hetero-oligomeric complexes and are present in most cells whereas expression of caveolin-3 is muscle-specific. Among caveolin proteins, CAV1α is indispensable for both the structure and function of caveolae. It is a 21–24 kDa integral membrane protein that possesses all the qualities of a scaffolding protein that functions to organize and concentrate specific lipids (e.g., cholesterol and sphingolipids) [9]–[11] and lipid-modified signaling molecules (e.g., G-proteins) [12], [13]. The contribution of cholesterol to caveolae vesicle formation has been clearly demonstrated [14], [15]. CAV1 contains an amphipathic helix that is inserted into the bilayer of caveolae. This helix also binds 13 cholesterol molecules and causes the raft-like lipid composition of caveolae [16]. In addition, the cavin-1 protein plays important roles in the stabilization of the caveolar domain and the induction of the membrane curvature of the caveolae pit [15], [17].

Caveolae are particularly abundant in adipocytes, endothelial cells, fibroblasts, and muscle cells. Functionally, caveolae are implicated in a variety of signal transduction-related events, transcytosis, membrane trafficking, and lipid homeostasis [18]–[20]. Of interest, it has been established that CAV1 exerts a negative regulatory effect on cell growth [21]. In human cancers, CAV1 expression is often lost, indicating that CAV1 possesses tumor suppressor capabilities [22]. In addition to cancer cells, some mammalian cell types, i.e. neurons, blood cells or lymphocytes, do not express any caveolin proteins, and therefore do not develop caveolar invaginations. Examination of systems from across the diversity of eukaryotes shows that caveolins appear late in eukaryotic evolution as they are restricted to most metazoans,vertebrate and invertebrate animals) [23]. These proteins are well-conserved from humans to nematodes [24]–[26] but are absent from flies. The lack of caveolin genes in the fungi (yeast) branch and protozoa establishes that the caveolar signaling pathway is a recent acquisition and that the mechanism of lipid-anchored proteins in these primitive organisms differs from higher eukaryotic cells.

Several independent lines of evidence suggest that CAV1 expression correlates with the generation of caveolae structures in cells. First, prototypical caveolae invaginations can be induced *de novo* on the surface of caveolin-negative mammalian cells upon heterologous expression of CAV1 [3], [27]–[29]. Second, deletion of the CAV1 gene from mice results in a complete absence of morphologically identifiable caveolae structures in all tissues and cell types that normally express CAV1 [30]. Third, recombinant expression of CAV1 in insect cells or *Escherichia coli* drives the formation of CAV1-rich vesicles [31], [32]. However, these studies did not document about the ability of these cells to internalize caveolar cargos.

Several questions remain concerning the minimal machinery required for caveogenesis and function of caveolae in endocytosis, and membrane trafficking. For example, it remains uncertain whether CAV1 alone is sufficient to generate functional caveolae in caveolin-negative mammalian cells upon expression of recombinant CAV1, or alternatively, if other proteins aid CAV1 to this process. To directly address this issue, we have exploited the protozoan parasite *Toxoplasma gondii* as a minimalist system for expression of caveolae structural proteins. The rationales of this choice are the following. First, *Toxoplasma* lacks plasma membrane domains with the characteristic features of caveolae, which is expected for a unicellular organism, and does not contain any caveolin or cavin genes. Second, *Toxoplasma* contains sphingolipids [33]–[35] and cholesterol [36], and has detergent-resistant membrane domains [37], [38], which suggests that the parasite can assemble lipid raft-like microdomains at the plasmalemma. Third, though *Toxoplasma* contains homologues coding for the (AP)2 complex and the light and heavy chains of clathrin, it does not express these proteins at the plasma membrane to form clathrin-coated pits [27]. Consequently, the absence of an operational clathrin-mediated endocytic pathway in *Toxoplasma* will facilitate the interpretation of data on caveolar ligand internalization on parasites recombinantly expressing caveolae structural proteins.

The aim of this study was to examine the ability of *Toxoplasma* to generate functional caveolae upon ectopic expression of caveolar proteins. We show constitutive formation of heterologous caveolae in the protozoan. However, *Toxoplasma* expressing either CAV1 or cavin-1, or expressing both CAV1 and cavin-1 are incompetent in internalizing caveolae-dependent cargos, indicating that CAV1 or cavin-1 are insufficient to develop functional caveolae at the parasite’s plasma membrane. However, in contrast to the single expressors, co-transfected *Toxoplasma* CAV1 and cavin-1 displays large entangled membranous structures that contain the two proteins, suggesting a collaborative role for CAV1 and cavin-1 to shape intracellular membranes.

## Materials and Methods

### Chemicals and Antibodies

All chemicals were obtained from either Sigma Chem. Co. (St. Louis, MO) or Fisher (Waltham, MA) unless indicated otherwise. Alexa Fluor 594-cholera toxin B (CT-B), BODIPY(red)-lactosylceramide (LacCer) and all secondary Alexa Fluor-coupled antibodies were obtained from Invitrogen (Eugene, OR). [1-^14^C]palmitic acid (sp act: 55 mCi mmol^−1^) was purchased from Amersham Corp. (Arlington Heights, IL). Primary antibodies used in this study were: rabbit monoclonal anti-human CAV1α (Epitomics, Inc, Burlingame, CA), rabbit polyclonal anti-human CAV1α provided by RGW Anderson (University of Texas Southwestern Medical Center), mouse monoclonal anti-GFP, mouse or rabbit monoclonal anti-HA (BD Biosciences, Mountain View, CA and Covance, Princeton State, NJ), mouse monoclonal anti-IMC1 provided by GE Ward (University of Vermont), mouse monoclonal anti-CPL provided by VB Carruthers (University of Michigan), and mouse polyclonal anti-ROP2.3.4 (three rhoptry proteins), GRA1 and SAG1 all provided by JF Dubremetz (University of Montpellier, France).

### Mammalian Cell Line, Culture Conditions and Parasite Propagation

The mammalian cell line used for the propagation of *Toxoplasma gondii* was primary human foreskin fibroblasts (HFF; commercial source: ATCC CRL-1635). HFF cells were grown as monolayers and cultivated in α-minimum essential medium (MEM) supplemented with 10% fetal bovine serum (FBS), 2 mM glutamine and penicillin/streptomycin (100 units/ml per 100 µg/ml) as described [39]. The tachyzoite RH strain (wild type, type I) of *Toxoplasma gondii* were used throughout this study. In addition, a parasite strain stably expressing GRASP55-RFP was used for Golgi localization studies (generous gift from VB Carruthers, University of Michigan). All tachyzoite strains of *T. gondii* were propagated in vitro by serial passage in HFF as described [40].

### Plasmid Construction for CAV1 Expression in *Toxoplasma*

*Stable transfection*: A *T. gondii* expression vector sagCATsag_TubFNRYFP (obtained from DS Roos, University of Pennsylvania) was used to generate *T. gondii* stably expressing copies of human CAV1 or derived-mutants. This vector facilitates the expression of recombinant proteins in *T. gondii* driven by the tubulin promoter with a C-terminal YFP fusion. Coding sequence of human CAV1 was PCR- amplified from pcDNA-CAV1, a plasmid harboring the ORF of human CAV1α generously given by RGW Anderson (University of Texas Southwestern Medical Center) using primers *f*-cav1 (ACTAGATCTGACAAAATGTCTGGGGGCAAATACGTAGACTC) and *r*-cav1 (TCACCTAGGTATTTCTTTCTGCAAGTTGATGCGGACATTGCTG). The resulting PRC fragment was digested with BglII and AvrII and ligated into the same restriction sites in sagCATsag_TubFNRYFP to obtain psagCATsag_CAV1-YFP.

For the construction of psagCATsag_CAV1^C133A^-YFP, two PCR were run using pcDNA-CAV1 as template, and first the primers *f*-cav1 and *r*-C133A (GGAAGCTCTTAATGGCTGGTACAACTGCCC), and second the primers *f*-C133A (GGGCAGTTGTACCAGCCATTAAGAGCTTCC) and *r*-cav1. The two PCR products were mixed and used for fusion PCR that was amplified with primers *f*-cav1 and *r*-cav1. The resulting PCR fragment was digested with BglII and AvrII and ligated into the equivalent restriction sites in psagCATsag_CAV1-YFP to generate psagCATsag_CAV1^C133A^-YFP. Using the same approach, psagCATsag_CAV1^C143A^-YFP and psagCATsag_CAV1^C156A^-YFP were engineered using the primers *f*-cav and *r*-C143A (GACACGGCTGATGGCCTGAATCTCAATC), *f*-C143A (GATTGAGATTCAGGCCATCAGCCGTGTC) and *r*-cav1, and the primers *f*-cav1 and *r*-C156A (CAAAGAGTGGGTCAGCGACGGTGTGG), *f*-C156A (CCACACCGTCGCTGACCCACTCTTTG) and *r*-cav1, respectively. CAV1 and mutant insertions were confirmed by enzymatic digestions and DNA sequencing. *Transient transfection:* The plasmid pHX-NTPHA was used to transfect *Toxoplasma* expressing caveolin or derived mutant with a N-terminal HA tag under the nucleoside triphosphatase III (NTPase) promoter. The genes of CAV1 or CAV1^C133A^ were cloned into the NheI and PacI restriction sites using the primers *f-*PHXcav1 (GAGAGCTAGCATGTCTGGGGGCAAATACGTA) and *r-*PHXcav1 (GCGCGCTTAATTAAATTTCTTTCTGCAAGTTGATG), and primers *f-*PHXcav1C133A (GATTGAGATTCAGGCCACCAGCCGTGTCTATTCCATCTACGTCCACACCGTCGCTGACCCACTC) and *r-*PHXcav1C133A (GAGTGGGTCAGCGACGGTGTGGACGTAGATGGAATAGACACGGCTGGTGGCCTGAATCTCAATC), creating the plasmids pHX-cav1 or pHX-CAV1^C133A^, respectively. Parasites were transfected with these plasmids by electroporation to ensure expression of CAV1 or CAV1^C133A^ as described [40].

### Plasmid Construction for Cavin-1 Expression in *Toxoplasma*

In order to express human cavin-1 in *T. gondii*, RNA was extracted from HFF cells and the coding sequence of human cavin was amplified by RT-PCR (SuperScript One-Step RT-PCR with Platinum Taq, Invitrogen) using primers *f*-cavin (ACTCCATGGAGGACCCCACGCTCTATATTGTCGAG) and *r*-cavin (TCATTAATTAATTATCACTAAGCGTAGTCTGGGACGTCGTATGGGTAGTCGCTGTCGCTCTTGTCCACCAGC). The RT-PCR fragment was digested with NcoI and PacI and directly ligated into the same sites of pNTP3-DHFR vector to generate pNTP3-cavin-DHFR. The reverse primer *r*-cavin contains the sequence of HA-tag, which allows the expression of cavin-1 with a C-terminal HA-tag. The plasmid pNTP3-DHFR facilitates the expression of recombinant proteins in *T. gondii* driven by the nucleoside triphosphate hydrolase promoter. The gene coding for dihydrofolate reductase included in the plasmid allows the selection of *T. gondii* stably expressing the recombinant protein under pyrimethamine exposure.

### Expression Analysis in *T. gondii* and Selection of Parasite Stable Lines

Expression plasmids containing the human CAV1, CAV1 mutants or human cavin-1 were multiplied in *E. coli* DH-5α and isolated using Qiagen Plasmid Maxi Kit. Parasites were transfected by electroporation using 50 µg plasmid DNA according to a published protocol [40]. For the generation of parasites stably expressing CAV1 or its mutants, parasites were transfected with corresponding plasmids, passaged in HFF cultures under the chloramphenicol (20 µM) selection until a stably growing parasite cell line is finally established. Likewise, the parasites stably expressing human cavin-1 was obtained by selecting with 2 µM pyrimethamine. For co-expression of CAV1 and cavin-1, the parasites transfected with sagCATsag_CAV1-YFP and stably expressing CAV1 parasites were transfected by electroporation using 50 µg of pNTP3-cavin-DHFR, and selected in both pyrimethamine and chloramphenicol.

### Immunoblot Analysis of Wild-type *T. gondii* or *T. gondii* Stably Expressing Either CAV1 or Cavin-1

For immunodetection on blots, wild-type and transgenic parasites were lysed by resuspension in SDS gel-loading buffer (50 mM Tris–HCl; pH 6.8), 50 mM 2-mercaptoethanol, 2% SDS, 0.1% bromophenol blue, 10% glycerol) followed by boiling in a waterbath. The samples (∼10^7^ parasites) were subjected to SDS-PAGE, and the proteins were then electrophoretically transferred to a membrane (Immobilon Transfer Membranes, Millipore, Bedford, MA). The membrane was immersed in blocking buffer (PBS containing 3% skim milk) for 60 min, and incubated with anti-GFP (1∶1000), anti-CAV1 (1∶20,000) or anti-HA (1∶2,000) antibodies in the blocking buffer for 60 min. Unbound antibody was removed by washing the membrane 6-times with blocking buffer. The membrane was then incubated with horseradish peroxidase-conjugated goat anti-mouse IgG antibody (Amersham Pharmacia Biotech; dilution, 1∶10000) in blocking buffer for an additional hour, before detection by chemiluminescence using ECL-Plus kit.

### Palmitoylation Assays

Intracellular parasites transiently expressing CAV1-HA were incubated in medium containing 1% FCS plus 0.2 mM [^14^C]palmitate bound to albumin (1% albumin, palmitate:albumin molar ratios of 2∶1) for 6 h, then either treated for 1 h at 37°C in MEM containing 20 mM HEPES (pH 7.4) before incubation for an additional hour at 37°C in buffer only. After parasite isolation from cells, caveolin proteins from [^14^C]palmitate-labeled parasites was immunoprecipitated with anti-HA antibodies as described [41] and immunoprecipitates were resolved in two duplicate SDS-PAGE gels. For demonstration of palmitoylation of caveolin, the gels were soaked in 1 M Tris (pH 7.5) or 1 M hydroxylamine (pH 7.5) for 14 h before being processed for fluorography.

### Detection of GM_1_

Liquid chromatography-mass spectrometry (LC-MS) was carried out using lipids from isolated *T. gondii* as described [42]. Briefly, cells were harvested, washed in PBS, and transferred to glass vials. Sphingolipid extracts, fortified with internal standards (*N*-dodecanoylsphingosine, *N*-dodecanoylglucosylsphingosine, and *N*-dodecanoylsphingosylphosphorylcholine, 0.5 nmol each), were prepared as described [43] and analyzed. The LC-MS consisted of a Waters Aquity UPLC system connected to a Waters LCT Premier orthogonal accelerated time of flight mass spectrometer (Waters, Millford, MA), operated in positive electrospray ionisation mode. Full scan spectra from 50 to 1500 Da were acquired and individual spectra were summed to produce data points each 0.2 s. Mass accuracy and reproducibility were maintained by using an independent reference spray by the LockSpray interference. The analytical column was a 100 mm×2.1 mm i.d., 1.7 µm C8 Acquity UPLC BEH (Waters). The two mobile phases were A: methanol:water:formic acid (74∶25:1); B: methanol:formic acid (99∶1), both containing 5 mM ammonium formate. A linear gradient was programmed–0.0 min: 80% B; 3 min: 90% B; 6 min: 90% B; 15 min: 99% B; 18 min: 99% B; 20 min: 80% B. The flow rate was 0.3 ml/min. The column was held at 30°C. Quantification was carried out using the extracted ion chromatogram of each compound, using 50 mDa windows. The linear dynamic range was determined by injecting standard mixtures. Positive identification of compounds was based on the accurate mass measurement with an error <5 ppm and its LC retention time, compared to that of a standard (±2%).

### CT-B Radiolabeling and Uptake

CT-B was radiolabeled with ^125^I (Amersham Corp.) by means of ICl [44] to yield a specific radioactivity of 286 cpm/ng protein for [^125^I]CT-B. For uptake assays by parasites and fibroblasts, 3×10^8^ extracellular wild-type and transgenic parasites (∼1 mg cell protein) or 3×10^6^ cultured HFF (∼1 mg cell protein) were incubated in serum-free MEM at 37°C with 0.1 µg/ml [^125^I]CT at different time points. One uptake assay using parasites was performed in medium with high concentration of K^+^ made of 142 mM KCl, 5 mM NaCl, 2 mM EGTA, 5 mM MgCl_2_, 25 mM Hepes-KOH and 1 mg/ml BSA (pH 7,2) at 37°C with [^125^I]CT to mimic the host cell cytosol ionic composition. After uptake, parasites and cells were transferred to 4°C and extensively washed 3 times with PBS containing 0.44 mM Ca^2+^, 3 times with PBS-Ca^2+^ supplemented with 1% BSA, and 3 times with PBS-Ca^2+^ to minimize non specific surface binding. After surface digestion by 0.1% pronase in MEM medium for 1 h, parasites and cells were harvested by centrifugation, briefly rinsed twice with PBS, lyzed in 0.01% Triton X-100 before determining radioactivity content for CT-B uptake.

### Fluorescence Microscopy Observations

Light and epifluorescence microscopy were performed on *Toxoplasma*-infected HFF seeded on sterile coverslips in 24-well culture dishes. Parasites expressing human CAV1 (and mutants) with a YFP tag were either observed live for direct YFP fluorescence or fixed for IFA as previously described (29) using anti-GFP (1∶100) or anti-CAV1 (1∶250) to analyze CAV1 localization. Parasites expressing human cavin-1 with a HA tag were fixed for IFA before incubation with anti-HA antibodies (1∶500). For dual or triple localization studies, these transgenic parasites were stained with anti-ROP2.3.4 (1∶100), anti-SAG1 (1∶500), anti-IMC1 (1∶100), anti-GRASP (1∶200), anti-GRA1 (1∶500), or anti-CPL (1∶500) antibodies. Slides were observed using a Nikon Eclipse E800 microscope equipped with a Spot RT CCD Camera and processed using Image-Pro-Plus software (Media Cybernetics, Silver Spring, MD) before assembly using Adobe Photoshop (Adobe Systems, Mountain View, CA). To visualize the caveolae-like structures in 3-D, intracellular parasites expressing CAV1 and/or cavin-1were fixed with 4% formaldehyde/0.02% glutaraldehyde/PBS and imaged with a fluorescent microscope (Nikon 90i) using a Plan-Apochromat 100×/1.4 NA. For each image, z-stacks were acquired and processed by applying a deconvolution algorithm using Volocity software (Improvision, Waltham, MA). To analyze the uptake of BODIPY-LacCer or Alexa Fluor 594-CT-B, parasites expressing CAV1-HA and CAV1-YFP, respectively, were used. They were harvested from HFF, passed through a 3-µm filter, washed twice with medium without serum, concentrated by centrifugation and resuspended in 100 µl medium without serum before exposure to fluorescent probes (final concentrations of 10 and 25 µM for LacCer and CT-B, respectively) at 37°C before washing with PBS and observed live under the microscope. Same parasite preparation was used for parasites expressing cavin-1-HA exposed to Alexa Fluor 594-CT-B except that after the pulse, the parasites were fixed for HA staining using Alexa Fluor 350. Parasite viability was verified by trypan blue exclusion assay.

### Electron Microscopy Studies

For thin-section transmission electron microscopy (EM), HFF infected with parasites expressing CAV1 and cavin-1 were fixed in 2.5% glutaraldehyde (Electron Microscopy Sciences; EMS, Hatfield, PA) in 0.1 M sodium cacodylate buffer (pH 7.4) for 1 h at room temperature, and processed as described [45] before examination with a Philips CM120 Electron Microscope (Eindhoven, the Netherlands) under 80-kV. For immunoelectron microscopy, intracellular parasites stably expressing caveolin were fixed in 4% paraformaldehyde (EMS) in 0.25 M HEPES (ph7.4) for 1 h at room temperature, then in 8% paraformaldehyde in the same buffer overnight at 4°C. They were infiltrated, frozen and sectioned as previously described [46]. The sections were immunolabeled with anti-GFP antibodies (1∶50), then with rabbit anti-mouse IgG antibodies, followed directly by 10 nm protein A-gold particles (Department of Cell Biology, Medical School, Utrecht University, the Netherlands).

### Protein Determination

Protein content was determined by the Bradford [47] assay, using serum bovine albumin (BSA) as a standard.

### Statistical Analysis

For comparison of values, *P* was determined using Student's *t*-test.

## Results

### Ectopic Expression of CAV1 by *Toxoplasma* Results in the Generation of Caveolae-like Structures

**Figure 1:**
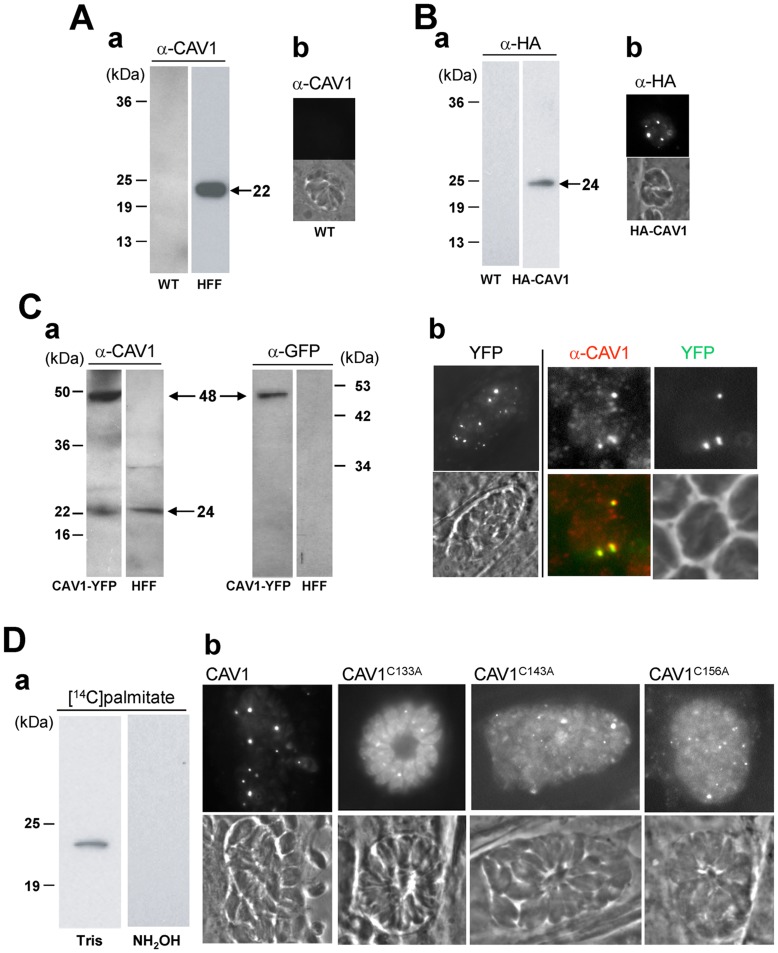
Expression and localization of recombinant CAV1 in *Toxoplasma*. (A) Panel a: Immunoblots of homogenates from wild-type *T. gondii* (left lane) and HFF (right lane) revealed with anti-CAV1 antibodies (α-CAV1), showing no expression of CAV1 by the parasites as confirmed by IFA in Panel b. (B) Expression of HA-CAV1 by transgenic parasites. Immunoblots of homogenates from wild-type *T. gondii* (left lane) and *Toxoplasma* transiently expressing HA-CAV1 for 36 h (right lane) revealed with anti-HA (α-HA), showing a band at ∼24-kDa in transgenic parasites. Panel b: Localization of HA-CAV1 in transgenic parasites by IFA showing a fluorescent punctuate pattern in transgenic parasites. (C) Panel a: Expression of CAV1-YFP by transgenic parasites. Immunoblots of homogenates from HFF infected with parasites stably expressing CAV1-YFP (left lanes) and uninfected HFF (right lanes) revealed with antibodies against CAV1 or against GFP (α-GFP) as indicated. The Western blots reveal bands at 48-kDa and 24-kDa corresponding to CAV1-YFP in *T. gondii* and CAV1 in HFF, respectively. Panel b: Localization of CAV1-YFP in transgenic parasites. Left: Direct fluorescence microscopy assays of CAV1-YFP-expressing parasites showing a vesicular staining. Right: Double immunofluorescence assays (IFA) of CAV1-YFP-expressing parasites using anti-CAV1 and anti-GFP antibodies illustrating structures co-labeled with the two antibodies. (D) Post-translational palmityolation of CAV1 in transgenic parasites. Panel b: Infected HFF with HA-CAV1-expressing parasites were labeled with [^14^C]palmitate for 6 h before isolation of the parasites. *Toxoplasma* homogenates were then immunoprecipitated with anti-HA antibodies and resolved in SDS gels containing either Tris or Tris/hydroxylamine for 12 h before analysis for fluorography. The gels shown are representative of one on two separate experiments, and revealed the association of radioactive palmitate with HA-CAV1. Panel b: Localization of mutant CAV1-YFP lacking palmitoylation residues in transgenic parasites. Direct fluorescence assays of intracellular *Toxoplasma* expressing CAV1^C133A^-YFP (CAV1^C133A^), CAV1^C143A^-YFP (CAV1^C143A^) or CAV1^C156A^-YFP (CAV1^C156A^) showing more cytosolic staining for YFP compared to *Toxoplasma* expressing wild-type CAV1.

As is characteristic of a unicellular organism, *Toxoplasma gondii* does not display caveolae structures on its surface. BLAST searches of the *Toxoplasma* database (www.ToxoDB.org) confirmed the absence of homologues coding for caveolins or cavins. To further verify the absence of proteins homologous to caveolins in *T. gondii*, parasite homogenates were assessed by immunostaining and Western blotting using antibodies against caveolin-1α (CAV1). No caveolin band was detected even after a long exposure to antibody or with low stringency washes of the blots whereas a 24-kDa band corresponding to the relative molecular weight of CAV1 was visible in homogenates from human fibroblasts (Figure 1A, panel a). Similarly, no signal for CAV1 on *Toxoplasma* lysates was apparent on immunoblots using the affinity-purified rabbit anti-CAV1 antibody (not shown). These observations were confirmed by the absence of signal by immunofluorescence assays (IFA) in parasites incubated with the same antibodies (Figure 1A, panel b).

Heterologous expression of CAV1 in caveolae-negative mammalian cells drives the restoration of functional caveolae [23], [27]–[29]. Overexpression of CAV1 induces the massive accumulation of caveolae-like vesicles in the cytosol of insect cells [31] and *E. coli* [32]. We examined whether the protozoan *Toxoplasma* would have the competence to form functional caveolae-like structures, namely to internalize caveolar ligands upon ectopic expression of mammalian CAV1. *Toxoplasma* were first transiently transfected with a plasmid containing the human CAV1 gene in fusion with the short HA tag at the N-terminus. Western blotting using anti-HA antibody showed an expected band at the molecular mass of CAV1 (Figure 1B, panel a). IFA show the presence of large fluorescent puncta within HA-CAV1-expressing parasites (Figure 1B, panel b).

To conduct detailed morphological studies, the entire ORF of human CAV1 was stably integrated into the genome of *T. gondii*. For this construct, a YFP tag was placed at the C-terminus of CAV1. Homogenates of fibroblasts infected with CAV1-YFP-expressing parasites were probed by Western blot using antibodies against either CAV1 or GFP. A band at 48-kDa was detected with both antibodies, which corresponds to the relative size of the chimeric protein expressed by the parasites (Figure 1B, panel a). Expectedly, a 24-kDa band of CAV1 was revealed by anti-CAV1 antibodies on blots of uninfected and infected fibroblast lysates. To ensure the viability of transgenic parasites, their replication rate was monitored and compared to wild-type parasites. Results show that the expression of CAV1 did not affect the time course of parasite infectivity as evaluated by counting the number of parasites per parasitophorous vacuole (PV) in infected cells or scrutinizing their morphology by EM (Figure S1). The localization of CAV1-YFP in transgenic parasites was analyzed by fluorescence imaging and by double IFA using anti-CAV1 and anti-GFP antibodies (Figure 1B, panel b). A dominant vesicular pattern was clearly discernible in the cytoplasm, and both CAV1 and GFP largely localized to the same structures.

Mammalian CAV1 has a long hydrophobic region followed by three cysteine residues that are palmitoylated and responsible for CAV1 association with the membrane of caveolae [41]. Palmitoyl groups are attached to cysteine residues by a thioester linkage and are then susceptible to hydrolysis by hydroxylamine. We tested whether CAV1 expressed by *Toxoplasma* undergoes palmitoylation, which would suggest membrane association. Infected fibroblasts were incubated with [^14^C]palmitate for 6 h, and radioactive parasites were then isolated from cells, lysed and HA-immunoprecipitated. Samples were resolved by SDS-PAGE gels containing either Tris buffer or hydroxylamine as a control before fluorography. Data show that [^14^C]palmitate was incorporated into CAV1 and that treatment with hydroxylamine removed radioactivity from the protein, confirming that the CAV1 expressed by *Toxoplasma* was properly palmitoylated (Figure 1A, panel c). These results were further confirmed by engineering three parasite cell lines expressing CAV1 in which each of the individual palmitoylated cysteine residues (C133, C143 and C156) was replaced by alanine residues. Parasites expressing Cav^C133A^-YFP, Cav^C143A^-YFP or Cav^C156A^-YFP were observed by fluorescence microscopy. The three mutant chimeric proteins were highly expressed, and distributed to the parasite cytoplasm and occasionally to small vesicles in contrast to wild-type CAV1 in which the pattern was predominantly vesicular (Figure 1C). This confirms that these cysteine residues of CAV1 are acylated by the parasite to mediate the incorporation of the protein into a membrane.

### Caveolae-like Structures in *Toxoplasma* are Cytoplasmic Vesicles with CAV1 Retained on the Limiting Membrane

**Figure 2:**
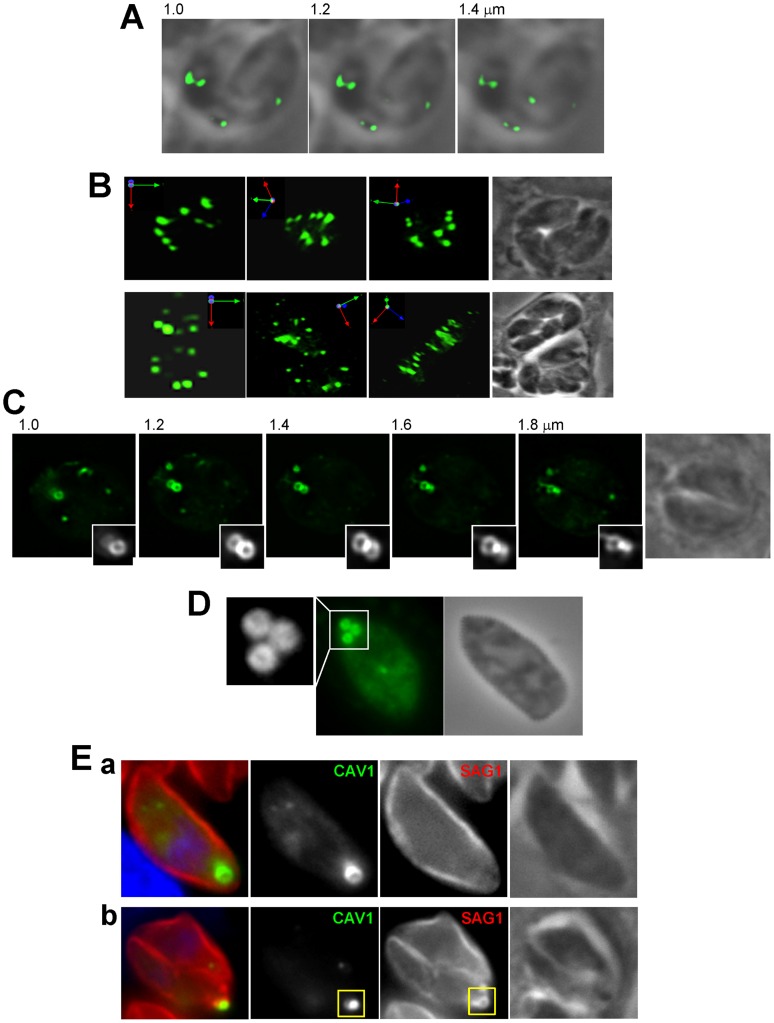
Morphological characterization of CAV1-containing vesicles in transgenic *Toxoplasma*. ( A–E) Direct fluorescence microscopy of intracellular *Toxoplasma* expressing CAV1-YFP illustrating the distribution of CAV1-containing vesicles within the parasite. (A) Images show individual z-slices from a z-series of fluorescence images. The images are shown with the fluorescence signal overlaying the corresponding phase contrast picture. (B) 3-D reconstructions of z-stacks of CAV1-labeled vesicles and rotated views of the images are shown. (C) Series of z-stacks of intracellular transgenic parasites revealing large CAV1-labeled vesicles with an annular staining pattern. (D) Series of z-stacks of extracellular transgenic parasites showing large CAV1-labeled vesicles with similar pattern as in C. (E) *Toxoplasma* expressing CAV1-YFP immunolabeled with antibodies against SAG1 (marker for the plasma membrane) show representative examples of CAV1-containing vesicles that are distant from the plasma membrane (panel a) or surrounded by the plasma membrane (panel b), which is forming residual body shown in the square.

We next wanted to provide more details about the abundance, morphology, and localization of the CAV1-containing structures in transgenic parasites. Overlaying phase contrast images with their corresponding fluorescent profiles in Figure 2A illustrated that the CAV1-containing structures were localized within the parasites. Quantification analysis to assess the distribution of these vesicles revealed that ∼45% were in the peri-Golgi region, ∼20% at the apical pole and ∼35% were located posteriorly behind the nucleus. Visualization of these structures in 3-D images reconstructed from optical z-sections illustrates that all transgenic parasites formed large fluorescent vesicles within the cytoplasm (Figure 2B). The number of vesicles per parasite varied between one (17%), two (39%), three (22%), four (19%) and five (3%) vesicles (n = 100 parasites). The fluorescence pattern of some of the large CAV1-containing structures looked annulated, which is indicative of a distribution of CAV1 on the limiting membrane of the vesicle as expected for a coat protein (Figure 2C and D).

As a prerequisite for functional studies on caveolar endocytosis in transgenic parasites, we looked for potential contact or insertion of CAV1-containing structures into the parasite’s plasma membrane. CAV1-YFP expressing parasites were immunostained with antibodies against SAG1, the major GPI-anchored protein of the plasma membrane which uniformly covers the entire parasite’s body. Microscopic observations of transgenic parasites did not show any association of CAV1-containing structures with the plasma membrane, or change in the general cytoarchitecture of this membrane (Figure 2E, panel a). In very rare occasions, the CAV1 fluorescent labeling was surrounded by the SAG1 staining but in this case the CAV1-associated structures were trapped in a parasite residual body (Figure 2E, panel a), which contains unused mother cell components that usually disappears after the completion of parasite division.

### CAV1 Localizes to and Near the Parasite Golgi

**Figure 3:**
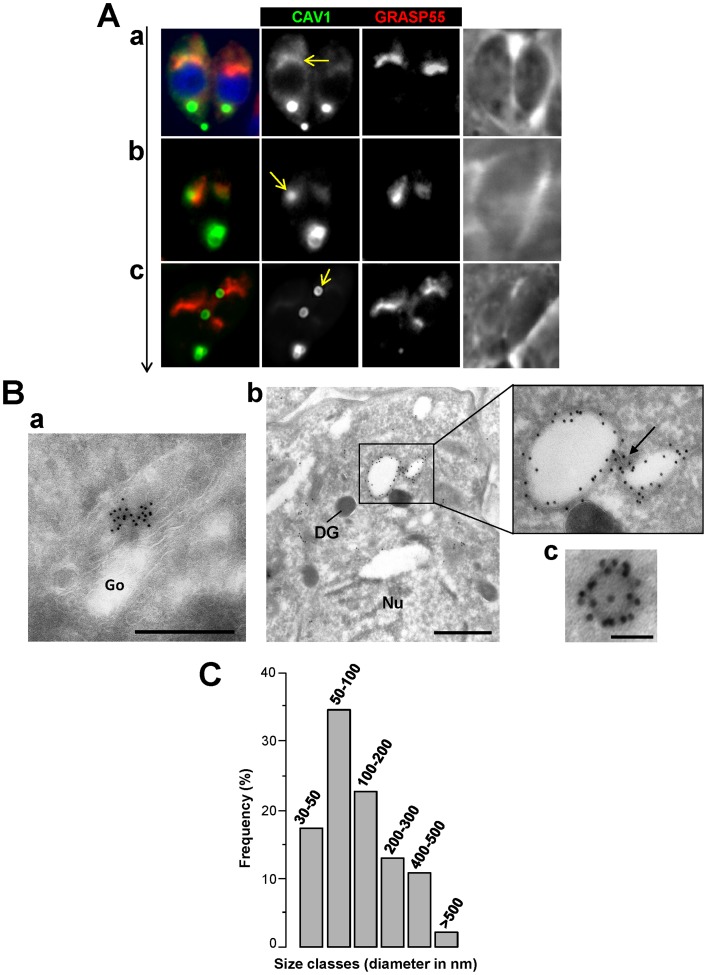
Spatial distribution of CAV1-labeled vesicles in the parasite Golgi area. (A) Fluorescence microscopy of *Toxoplasma* expressing CAV1-YFP and GRASP55-RFP (marker for the Golgi) showing CAV1-staining (arrows) on and near the Golgi complex that is apical to the nucleus (DAPI-stained). Vertical arrow from panel a to c shows increased levels of expressed CAV1 and association with vesicles. (B) ImmunoEM staining of intracellular *Toxoplasma* expressing CAV1-YFP using anti-GFP antibodies revealed by 10 nm-protein A-gold particles showing a staining on the Golgi (panel a) and peripheral staining of large (panel b) and small (panel c) vesicles in the cytoplasm. The arrow shows a membranous structure connecting two vesicles. DG, dense granule; Nu, nucleus. Bars are 400 and 40 nm in panels a and b, respectively. (C) Distribution of sizes of CAV1-containing vesicles labeled with anti-GFP antibody-protein A gold particles. The diameters in nm of over 100 gold-labeled vesicles were measured from 40 parasite sections taken at 66,000× magnification, and their frequency was tabulated.

In mammalian cells, CAV1 is synthesized in the ER, shipped to the Golgi as detergent-soluble oligomers that associate with lipid raft domains [48], and is palmitoyated for membrane attachment to vesicles that bud from the Golgi. In due course, these pre-caveolae vesicles traffic to the plasma membrane for insertion [49]. Since mammalian CAV1 was successfully expressed by *Toxoplasma* and the resulting protein was post-translationally modified by palmitoylation (Figure1D), we next examined the localization of CAV1 with respect to the Golgi in the parasite. We transfected CAV1-YFP into a parasite line expressing a fluorescent version of the Golgi marker GRASP55 and observed the parasites by microscopy. *Toxoplasma* is a polarized cell with a single Golgi apparatus apical to the nucleus (Figure 3A). CAV1-YFP showed a diffuse fluorescent staining in the anterior region of the parasite that partially overlapped with the GRASP55-RFP labeling on the Golgi stacks (Figure 3A, panel a). Treatment of extracellular CAV1-YFP-expressing parasites with brefeldin A (5 µg/ml for 30 min), a drug affecting the activation of Arf1, resulted in a bright YFP signal in the endoplasmic reticulum (data not shown). In some parasites, a more prominent CAV1-YFP signal was observed adjacent to the Golgi apparatus (Figure 3A, panel b). In other parasites, vesicles with well-defined CAV1 peripheral staining were detected in the post-Golgi area (Figure 3A, panel c). To analyze the localization of CAV1 in finer detail, we performed immunoEM using anti-GFP antibodies on CAV1-YPF-expressing parasites. Clusters of gold particles were seen on Golgi stacks of parasites (Figure 3B, panel a). Gold particles were also highly concentrated at the limiting membrane of electron-lucent vesicles located in the post-Golgi region (Figure 3B, panels b and c). This peripheral immunostaining of large vesicles was compatible with the taurus-shape of the CAV1-positive structures observed by fluorescence microscopy (Figures 2C and 3A, panel c). Sometimes these vesicles were connected to each other with thin membrane projections, suggestive of a dynamic vesicular system (see arrow in Figure 3B, panel b). Quantitative morphological analysis indicates that transgenic parasites equally drove the formation of caveolae-sized structures (<100 nm in diameter; Figure 3C) and of particularly large caveolae-like vesicles (>100–500 nm in diameter).

In mammalian cells, caveolae are ampulliform invaginations of the plasma membrane with a diameter in the range of 50–100 nm [50]. The morphology of the plasma membrane of transgenic parasites was also scrutinized in detail. No invaginations of the plasma membrane resembling caveolae pits could be identified by immunogold staining. Altogether, these qualitative data suggest that CAV1 is expressed in the Golgi and incorporated into vesicles of different sizes originating from this organelle before randomly distributing within the cytoplasm of transgenic parasites.

### After Biogenesis, CAV1-containing Structures do not Intersect with any Organelles Specific for *Toxoplasma*

**Figure 4:**
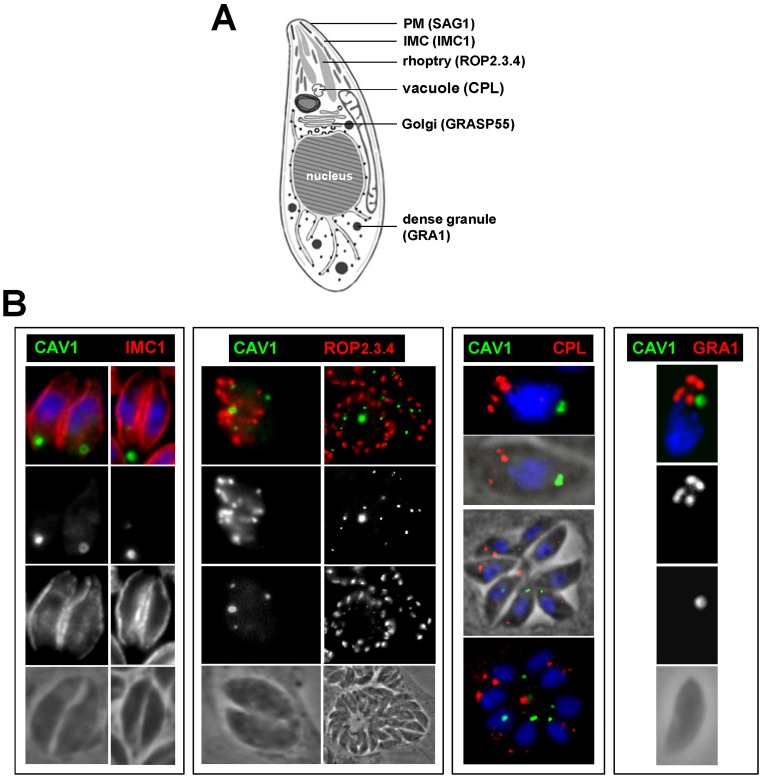
Distribution of CAV1-labeled vesicles relative to organelles specific to *Toxoplasma*. (A) Schematic representation of *Toxoplasma* structures with their markers. (B) Fluorescence microscopy of *Toxoplasma* expressing CAV1-YFP immunolabeled with antibodies against IMC1 (marker for the IMC), ROP2,3,4 (marker for the rhoptries), CPL (marker for the vacuole) and GRA1 (marker for dense granules) showing no overlap between CAV1-containing vesicles and any known organelles. These images are representative of the 35–50 PVs viewed.

The association of CAV1-YFP with the parasite’s Golgi suggests that vesicles containing CAV1 are formed *de novo* by budding from the Golgi complex as has been described in mammalian cells. To rule out the possibility that other pre-existing organelles in the parasite are involved in forming CAV1-containing structures or have incorporated CAV1 into their membrane, we analyzed the distribution of CAV1-YFP relative to protein markers for different organelles of *Toxoplasma* (as shown in the schematic representation of the *Toxoplasma* cell in Figure 4A). In particular, we probed the localization of CAV1-YFP with respect to the inner membrane complex, rhoptries, vacuolar compartments (VAC) and dense granules (Figure 4B).

*Toxoplasma* is delimited by a trilaminar pellicle, which is composed of the plasma membrane tightly associated with flattened membrane cisternae forming the inner membrane complex (IMC) [51]. The IMC is involved in parasite motility. Structurally, it is continuous along the length of the parasite with some small local interruptions permitting exocytic events and is absent from the basal pole of the parasites. Because of its localization beneath the parasite’s plasma membrane, we looked for any possible connectivity between CAV1-containing structures and the IMC. We analyzed the localization of CAV1-YFP and the protein IMC1, which is a major structural component of the IMC in *Toxoplasma* [52]. No physical association was observed between CAV1-containing structures and the membranes forming the IMC as stained for IMC1 (Figure 4B).

Rhoptries are apical secretory organelles involved in parasite invasion. Because precursors of rhoptries are formed in the Golgi complex and, like caveolae vesicles, are cholesterol-enriched [36], [53], we scrutinized the distribution of CAV1-containing structures relative to the rhoptry organelles stained for three major proteins, ROP2, ROP3 and ROP4. Although the CAV1-containing structures were sometimes very close to the basal end of the rhoptries, they remained physically distinct compartments (Figure 4B).

*Toxoplasma* possesses unique vacuolar compartments named VAC with properties of mammalian late endosomes [54], [55]. Since VAC organelles lie at the intersection of the exocytic and endocytic networks, we next looked at possible co-localization of CAV1 with CPL, a resident enzyme of VAC organelles. No overlap was observed between the CPL and CAV1 signals, indicating that VAC and CAV1-containing structures are independent organelles (Figure 4B).

Dense granules are secretory vesicles important for the parasite's intracellular lifestyle [56]. They are 200-nm electron dense organelles that localize throughout the parasite and are packed with proteins such as GRA1. Several studies support that dense granules constitute the default pathway for protein secretion in *T. gondii*, suggesting that specific signals are required for targeting to other secretory organelles like rhoptries. Proteins lacking proper targeting signals - such as foreign proteins expressed by *Toxoplasma* - are deviated to dense granule pathway for exocytosis (elimination) in the PV [57], [58]. Because of similarities in the size and random distribution in the cytoplasm of the dense granules and CAV1-containing structures, as well as the involvement of dense granules in the expulsion of unwanted proteins, we examined whether CAV1 could be part of the dense granule pathway. No intersection was observed between CAV1-containing structures and dense granules stained for GRA1 (Figure 4B), indicating that CAV1 is neither incorporated into dense granules nor released into the PV. Instead CAV1-containing structures are retained by the parasites and generated at each cycle of parasite replication. This suggests that the sequence motifs and scaffolding domains of human CAV1 are recognized by the biosynthetic machinery of *Toxoplasma*, leading to novel compartments with morphological properties shared with mammalian pre-caveolae organelles.

### CAV1-expressing *Toxoplasma* is Unable to Internalize Caveolar Ligands

**Figure 5:**
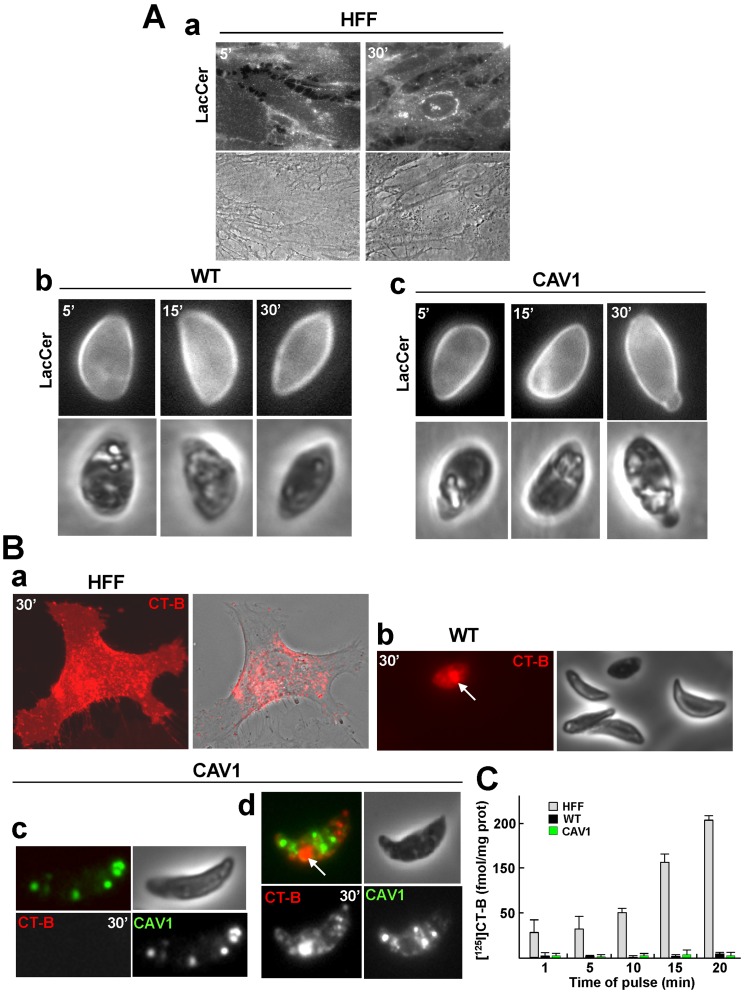
Functional studies for caveolar endocytosis on CAV1-expressing *Toxoplasm*a. (A) Assessment by live fluorescence microscopy of the intracellular distribution of BODIPY-LacCer in HFF (panel a), extracellular wild-type parasites (panel b) and extracellular HA-CAV1-expressing *Toxoplasm*a (panel c) at the indicated pulse times. (B) Same assay using Alexa Fluor 594-CT-B on HFF (panel a), extracellular wild-type parasites (panel b) and extracellular CAV1-YFP-expressing *Toxoplasm*a (panel c-d). To discriminate between dead and live parasites, trypan blue exclusion assays were performed. Trypan blue-positive parasites are indicated by an arrow in panels b and d). (C) Quantitative assays to monitor the kinetics of endocytosis of CT-B by wild type *Toxoplasm*a, CAV1-expressing parasites and fibroblasts. Extracellular parasites or monolayers of fibroblasts were incubated at 37°C for the indicated pulse times with in serum-free medium supplemented with [^125^I]CT-B prior to washing and surface-protease digestion at 4°C. Pronase-resistant values were normalized to the cell protein content in each lysate. Data, expressed in fmol/mg cell protein, are means ± S.D. of 3 independent assays for parasites or fibroblasts.

Our morphological observations fail to detect the presence of caveolae-like invaginations at the plasma membrane of transgenic *Toxoplasma*. However, these structures may be present at very low concentrations or have an unusual cycle of formation or activity, making caveolar spots on the parasite’s surface difficult to identify by light or electron microscopy. We next tested whether transgenic parasites could drive the internalization of well-known caveolae ligands. Glycosphingolipids and their derivative analogs such as lactosylceramide (LacCer) are selectively internalized via caveolae in mammalian cells [59]. We added BODIPY-labeled LacCer to the medium to examine the possible internalization of this caveolar ligand by CAV1-expressing *Toxoplasma.* We used human fibroblasts and wild-type *Toxoplasma* as positive and negative controls, respectively. Fibroblasts exposed to BODIPY-LacCer at 37°C showed a peripheral punctate staining pattern after a 5 min pulse and perinuclear staining corresponding to the Golgi apparatus after 30 min (Figure 5A, panel a). By contrast, in extracellular wild-type *Toxoplasma*, BODIPY-LacCer was uniformly distributed at the surface after a 5, 15 or 30 min pulse (Figure 5A, panel b). HA-CAV1-expressing parasites also showed a staining pattern restricted to the surface without any evidence of LacCer internalization (Figure 5A, panel c).

In mammalian cells, the B-subunit of cholera toxin (CT-B) binds to the ganglioside GM_1_ at the plasma membrane and is internalized by caveolar endocytosis [60], [61]. We wanted to exploit CT-B as another marker of caveolae to monitor the potential internalization of the toxin into transgenic parasites. Our mass spectrometry analysis of lipids extracted from axenic parasites has shown that *Toxoplasma* contains the ganglioside GM_1_; however, the localization of this lipid is not known (Figure S2). Human fibroblasts incubated at 37°C for 30 min with Alexa Fluor 594-CT-B exhibited fluorescence staining of the plasma membrane and in internal vesicles (Figure 5B, panel a). We exposed extracellular wild-type *Toxoplasma* to the fluorescent toxin under the same conditions. To ensure the viability of the parasite in the presence of the toxin, trypan blue exclusion assays were performed after the pulse and just prior to live microscopy. Live parasites did not bind or internalize Alexa Fluor 594-CT-B as no fluorescence signal was detected on any parasites from 3 independent assays (Figure 5B, panel b). However, trypan blue-stained parasites were positively labeled for CT-B. These parasites represented less than 1–3% of total parasite population. The CT-B labeling in dead parasites was predominantly localized to the central region and likely corresponded to the parasite’s Golgi, the biosynthetic site for gangliosides. In this case, CT-B would be retained on the Golgi through its affinity for GM_1_ after penetration into parasites with ruptured plasma membranes. When extracellular CAV1-YFP-expressing parasites were incubated with labeled CT-B, no fluorescence was associated with live parasites or within CAV1-containing structures as observed from 3 independent assays (Figure 5B, panel c). In dead transgenic parasites, CT-B accumulated in an area resembling the Golgi as well as in smaller vesicles (Figure 5B, panel d). These vesicles did not correspond to CAV1-containing structures since these latter were never positive for CT-B. Thus, even if parasites were alive during the pulse with CT-B, CAV1-containing structures were not implicated in CT-B internalization and transport to the Golgi.

Finally, we chose a more sensitive approach by performing radioactive uptake assays to assess the presence or the absence of CT-B inside transgenic parasites. Fibroblasts or extracellular parasites were incubated at 37°C from 1 to 20 min with radioiodinated CT-B, then washed, acid-stripped to remove surface-bound ligand and counted for radioactivity. Figure 5C shows the kinetics of [^125^I]CT-B accumulation. Expectedly, fibroblasts internalized CT-B with high efficiency. By contrast, no significant values for CT-B uptake were observed in *Toxoplasma*, either wild-type or CAV1-expressing parasites. In parallel, CT-B uptake assays were performed in medium mimicking intracellular fluid conditions (high K^+^ and low Na^+^ concentrations) to reproduce a more physiological environment for intracellular (metabolically active) *Toxoplasma*. No significant [^125^I]CT-B accumulated in the parasite fractions. These data indicate that CAV1 expressed by *Toxoplasma* does not drive the internalization of surface-bound caveolar ligands in accordance with the lack of invaginated caveolae at the plasma membrane.

### Cavin-1 Expressed by *Toxoplasma* Localizes to Vesicles, the Cytosol and the Nucleus

In mammalian cells, cavin proteins are involved in the regulation of caveolae assembly, and modulate the function of caveolins by promoting the membrane remodeling and trafficking of caveolin-derived structures [17], [62]. As with *caveolins*, the *Toxoplasma* genome does not contain any *cavin* homologues. Our hypothesis is that the absence of caveolar endocytic activities upon expression of CAV1 alone in *Toxoplasma* may be due to the absence of scaffolding proteins of caveolae such as cavins in transgenic parasites. Upon dual expression of CAV1 and cavin-1, the parasites may then be able to develop functional caveolae.

**Figure 6:**
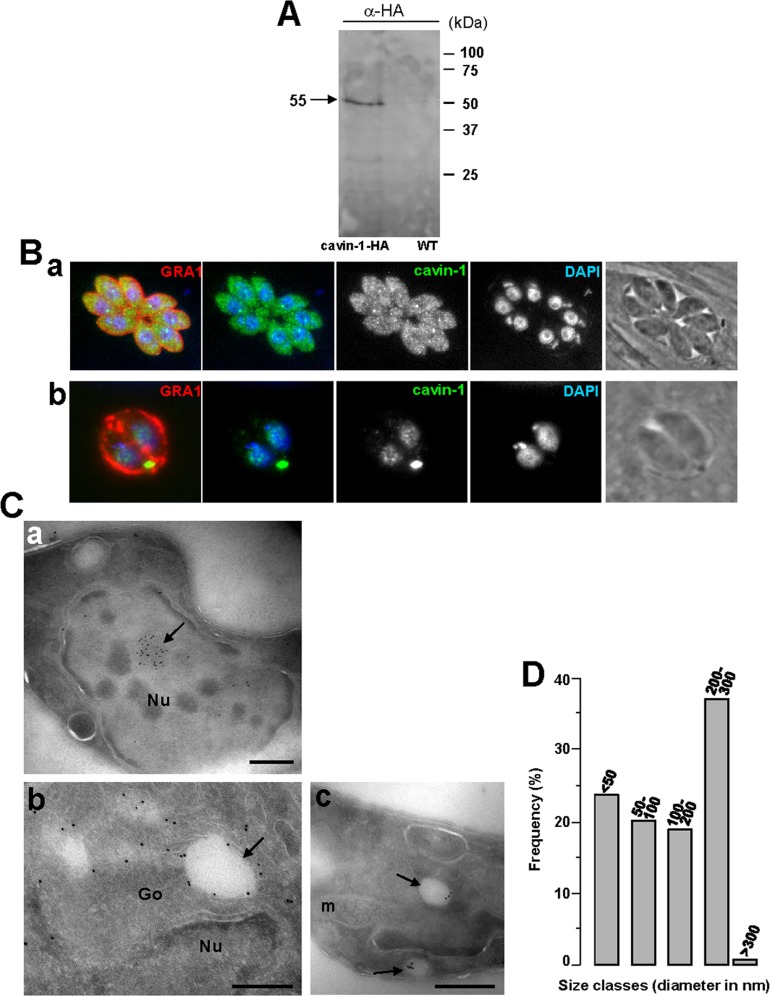
Expression and localization of cavin-1 in *Toxoplasma*. (A) Expression of cavin-1-HA by transgenic parasites. Immunoblots of homogenates from wild-type *T. gondii* (left lane) and *Toxoplasma* stably expressing cavin-1-HA (right lane) revealed with anti-HA antibodies (α-HA). The Western blot shows a band at ∼55-kDa in transgenic parasites. (B) Localization of cavin-1-HA in transgenic parasites. IFA assays of cavin-1-HA-expressing *Toxoplasma* using anti-HA antibodies showing staining in vesicles (panels a and b), the cytosol (panel a) and the nucleus (DAPI; panel b). (C) Ultrastructure of cavin-1-containing structures formed in transgenic *Toxoplasma*. ImmunoEM staining of intracellular *Toxoplasma* expressing cavin-1-HA using anti-HA antibodies revealed by 10 nm-protein A-gold particles confirming a staining within the nucleus (panel a), the cytosol (panel b) or on vesicles in the Golgi area (Go) (arrow in panel b) and vesicles randomly distributed in the cytoplasm (arrows in panel c). Arrow shows a membranous structure connecting two vesicles. m, mitochondrion; Nu, nucleus. Bars are 200. (D) Size distribution of cavin-1-containing vesicles labeled with anti-HA antibodies-protein A gold particles. The diameters in nm of over 70 gold-labeled vesicles were measured from 50 parasite sections taken at 66,000× magnification, and their frequency was tabulated.

**Figure 7:**
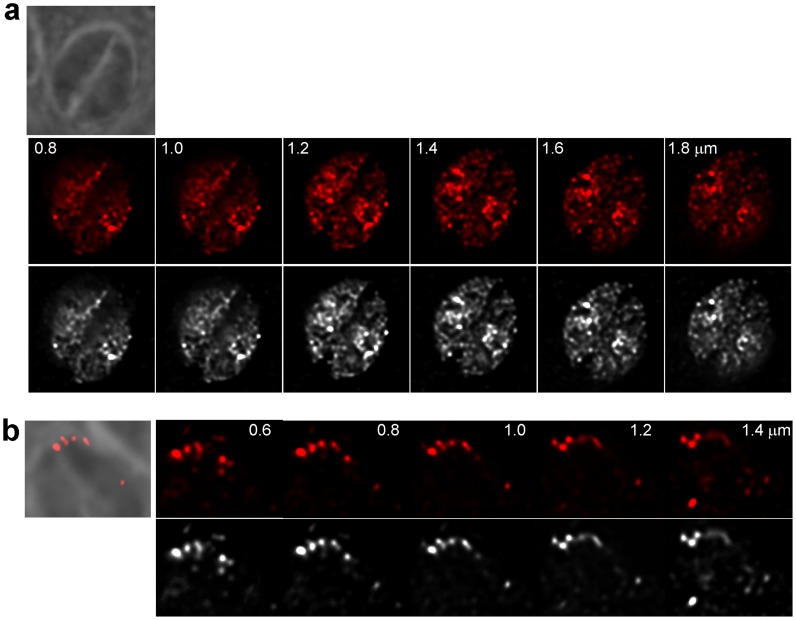
Morphological characterization of cavin-1-associated strucures in transgenic *Toxoplasma*. IFA on *Toxoplasma* expressing cavin-1-HA using anti-HA antibodies. A series of z-stacks are shown on parasites with a nuclear staining (panel a) or a vesicular staining at the cell periphery as demonstrated by the overlay of fluorescence with the corresponding phase contract images.

**Figure 8:**
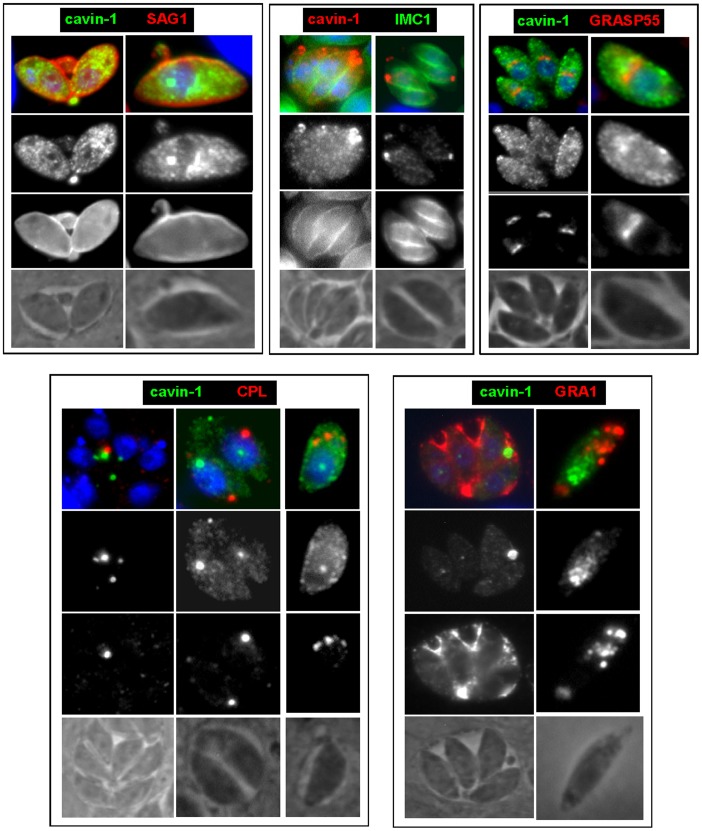
Distribution of cavin-1-labeled vesicles relative to other structures in *Toxoplasma*. Fluorescence microscopy of *Toxoplasma* expressing CAV1-YFP immunolabeled with antibodies against SAG1 (for the plasma membrane), IMC1 (IMC), GRASP55 (Golgi), CPL (vacuole) and GRA1 (dense granules).

We first engineered a stable strain of *Toxoplasma* expressing human cavin-1 fused with the short HA tag at the C-terminus. Western blotting using anti-HA antibody showed a band at the molecular mass of 50-kDa as expected for cavin-1 (Figure 6A). We next were interested in determining the localization of cavin-1 in the transgenic parasites. In mammalian cells, besides their association with caveolae, cavin-1 has other localizations such as the cytosol and the nucleus. This protein has a putative nuclear localization sequence [63] and can be found in the nucleus where it is involved in nuclear transcriptional and regulatory functions. This localization is typical for young and quiescent cells. During senescence, though, cavin-1 is concentrated in the cytosol and the plasma membrane [64]. Comparatively, IFA on cavin-1-expressing *Toxoplasma* revealed various localizations for cavin-1, and often the same parasite displayed different sites containing cavin-1 (Figure 6B). The predominant localization for cavin-1 was its association with different size vesicles in the cytoplasm (∼80% of parasites; Figure 6B, panels a and b). In many parasites, cavin-1 distributed uniformly to the cytoplasm (∼60% of parasites Figure 6B, panel a) and few of them showed cavin-1 expression in the nucleus as demonstrated by an overlap between the DAPI and HA staining (Figure 6B, panel b). ImmunoEM on cavin-1-HA-expressing parasites confirmed the localizations of cavin-1 that were intranuclear (Figure 6C, panel a), cytosolic (Figure 6C, panel b) and vesicular (Figure 6C, panels b and c). Quantitative morphological analysis based on immunogold-labeled parasites indicates that transgenic parasites formed cavin-1-containing structures of various sizes from 50 to 300 nm in diameter (Figure 6D). Fluorescence microscopic visualization of these organelles in 3-D reconstructions of optical z-stack sections illustrated a staining of cavin-1-HA inside the nucleus, within patches in the nucleoplasma (Figure 7, panel a). When cavin-1-HA is associated with vesicles, some of these structures were seen aligned near the plasma membrane as revealed by the overlay of the corresponding phase contrast image to the fluorescent profiles (Figure 7, panel b). The number of cavin-1-HA vesicles varied from one to 5 per parasite, and their morphology was distinct from the CAV1-containing vesicles. To examine whether cavin-1-containing vesicles are derived from preexisting membranes or organelles, or are formed *de novo* by transgenic parasites, we performed co-staining assays with various parasite markers (Figure 4A). Our microscopic observations shown in Figure 8 did not reveal any association of cavin-1 with the plasma membrane, the IMC, the Golgi complex, the VAC compartment or dense granules, indicating that cavin-1-containing vesicles are formed *de novo* and never intersect with any of these parasite structures after biogenesis.

### *Toxoplasma* Co-expressing CAV1 and Cavin-1 Restructure CAV1- and Cavin-1-Containing Vesicles into Large Membranous Tubules

**Figure 9:**
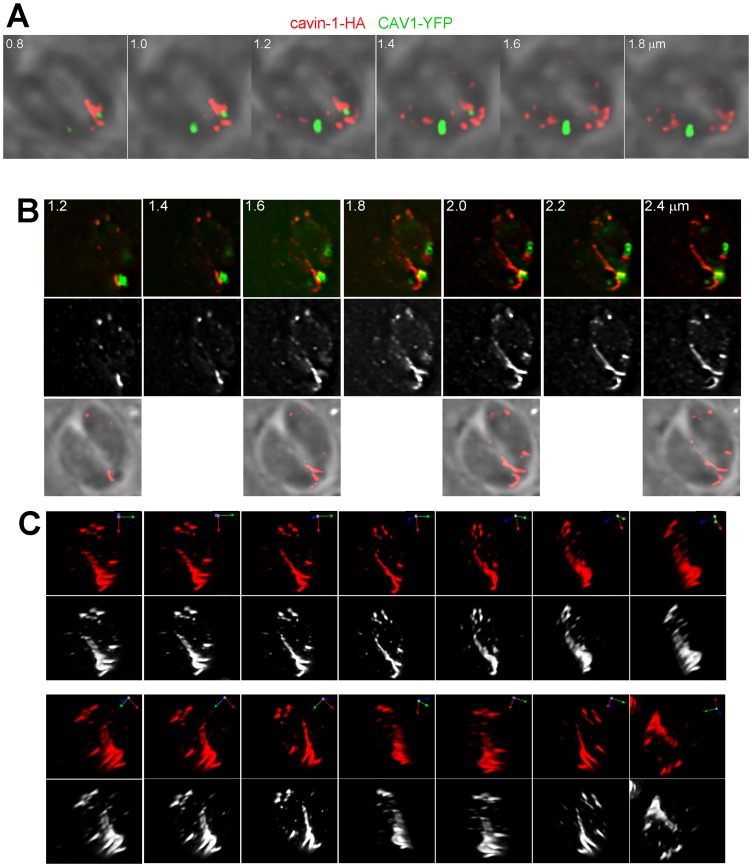
Morphology of cavin-1-and CAV1-positive structures in *Toxoplasma* co-expressors. (A–C) Fluorescence microscopy of transgenic parasites expressing cavin-1-HA and CAV1-YFP showing several cavin-1-and CAV1-labeled vesicles and tubules at the parasite periphery. Anti-HA antibodies were used to detect cavin-1-HA. (A) Individual z-slices of fluorescent signals (CAV1 and cavin-1) overlaid with the corresponding phase contrast images. (B) Individual z-slices of a co-expressing parasite showing either merged images of CAV1 and cavin-1, cavin-1 alone or cavin-1 staining overlaid with the corresponding phase contrast images. (C) 3D-reconstructions of the optical z-stack acquired from the parasite in (B). Multiple rotated views of the 3D images are shown.

**Figure 10:**
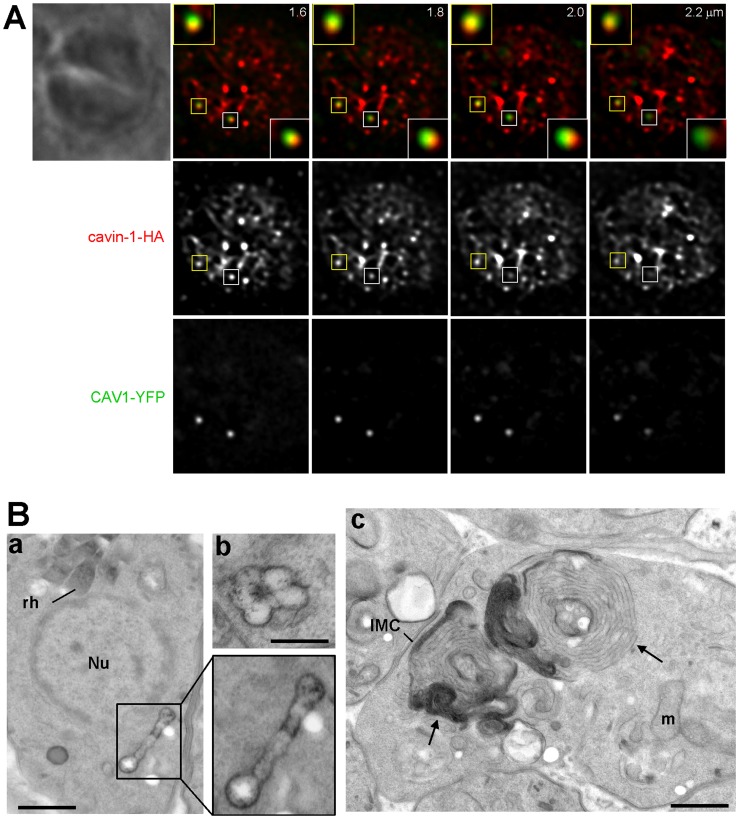
Co-distribution of CAV1-labeled vesicles and cavin-1-containing structures in transgenic parasites. (A) Fluorescence microscopy of transgenic parasites co-expressing CAV1-YFP and cavin-1-HA. Anti-HA antibodies were used to detect cavin-1-HA. Series of z-slices of transgenic parasites showing a close proximity of CAV1-YFP-labeled vesicles to cavin-1-containing structures (red) as illustrated in the insets. (B) Transmission EM of co-transfected parasites showing the presence of large neoformed membrane structures, tubules (panel a), vesicles (panel b) and whorls (panel c, arrows) in the parasite cytoplasm (arrows). m, mitochondrion. Bar is 200 nm. IMC, inner membrane complex; Nu, nucleus; rh, rhoptries. Bar is 200 nm.

**Figure 11:**
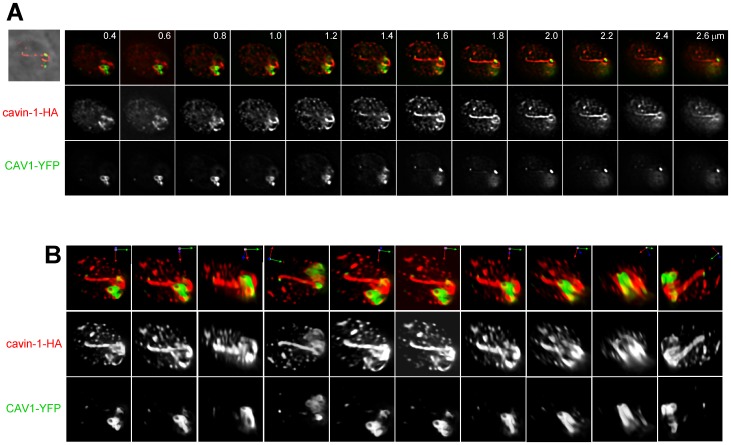
Interaction of CAV1-labeled structures with cavin-1-induced tubules in transgenic parasites. Fluorescence microscopy of transgenic parasites co-expressing CAV1 and cavin-1. (A) A series of z-slices of two co-expressing transgenic parasites. Shown are phase contrast images overlaid with the corresponding fluorescence signals, merged images displaying CAV1 and cavin-1 fluorescence and images with only CAV1 or cavin-1 fluorescence shown. (B) 3D-reconstructions of z-stacks from the transgenic parasites in (A) shown in multiple rotated views.

**Figure 12:**
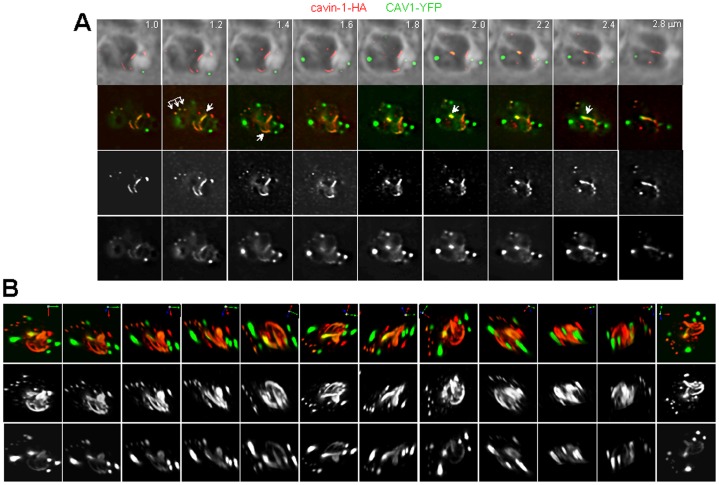
Alignment of CAV1-labeled vesicles with cavin-1-induced tubules in transgenic parasites. (A–B) Fluorescence microscopy of transgenic parasites co-expressing CAV1 and cavin-1. In B: series of z-stacks of transgenic parasites showing physical connection between the fluorescence pattern of structures containing CAV1 and cavin-1.

Parasites expressing cavin-1 were next transfected with the plasmid carrying CAV1 tagged with YFP at its C-terminus, in an attempt to select a parasite line stably expressing both caveolar proteins. However, simultaneous expression of CAV1 and cavin-1 was poorly tolerated by the parasites, leading to the loss of either expression. Because CAV1 is the major structural component of caveolae and cavin-1 only assists CAV1 to stabilize caveolae, we performed our experiments on parasites stably expressing CAV1 with the semi-stable expression of cavin-1 to yield ∼20% of double transfectants. The morphology of CAV1- and cavin1-labeled structures in double expressors was inspected by fluorescence microscopy. Dual expression of CAV1 and cavin-1 by *Toxoplasma* led to a spatial reorganization, and a dramatic change in the size and shape of structures containing these two proteins as illustrated in Figures 9,10,11,12 and Figure S3. Figure 9A shows the abundance of cavin-1-labeled structures in the cytoplasm of two parasites co-expressing CAV1 and cavin-1 as compared to *Toxoplasma* expressing cavin-1 alone (Figures 6,7,8). These structures had various shapes and sizes with some of them up to 700 nm in length. No staining was visible inside the parasite’s nucleus. More spectacularly, Figure 9B–C shows that co-expression of CAV1 and cavin-1 also induced the formation of very long membrane tubules that were positive for cavin-1. The extensive tubulation of the cavin-1 structures was further demonstrated in 3-D reconstructions of z-stacks shown in a rotated view in Figure 9C. The long tubule exhibited branched structures at one end of the parasite. Overlaying phase contrast images with the corresponding fluorescent profiles for cavin-1 illustrates that cavin-1-containing tubules run parallel to the parasite’s body and are aligned with the pellicle. The structures containing CAV1 were also increased in number and volume, and had a more cylindrical than spherical shape as compared to CAV1 single expressors.

### Despite Interconnection between the Structures Containing CAV1 and Cavin-1, *Toxoplasma* Remains Inaccessible to Caveolar Cargos

We next analyzed the relative distribution of CAV1- and cavin1-labeled structures in double expressors. Detailed analysis of z-stacks of the parasites shown in Figure 9B revealed a close proximity of structures containing CAV1 and cavin-1. In one parasite shown in Figure 10A, two vesicles harbored a positive signal for the two proteins. This may suggest a co-distribution of CAV1 and cavin-1 on the same vesicle as observed in mammalian cells in which CAV1 and cavin-1 co-localize on caveolae at the plasma membrane [62]. We performed transmission EM to inspect the ultrastructure of co-transfected parasites with CAV1 and cavin-1. In the cytoplasm of double expressors, we observed an accumulation of newly formed tubular and vesicular membranous structures without physical connection with any intracellular membrane of pre-existing organelles or association with the plasma membrane (Figure 10B). Some structures formed very long and convoluted membrane whorls (Figure 10B, panel c), indicating that the co-expression of CAV1 and cavin-1 imposed a dramatic structural change in the endo-membrane system of *Toxoplasma*. The close vicinity (∼50–100 nm) of CAV1- and cavin-1-labeled structures (vesicles or tubules) was observed in ∼80% of co-expressers while the co-distribution of CAV1 and cavin-1 on the same structures was noticeable in ∼30% of the parasites. More impressively, about 10% of the parasites displayed an elaborate and tangled network of membranous structures containing CAV1 and cavin-1 as documented on rotated views of serial sections of the parasites (Figure 10). Several CAV1- and cavin-1-positive structures were either closely intertwined with each other or co-aligned in the same area of the parasite (Figure 12 and Figure S3). These observations suggest that CAV1 and cavin-1 have a synergistic effect to shape membranes. These two proteins may be recruited to the same membrane domains and, cavin-1 may be involved in the stabilization of the CAV1-containing structures, as described in mammalian cells.

**Figure 13:**
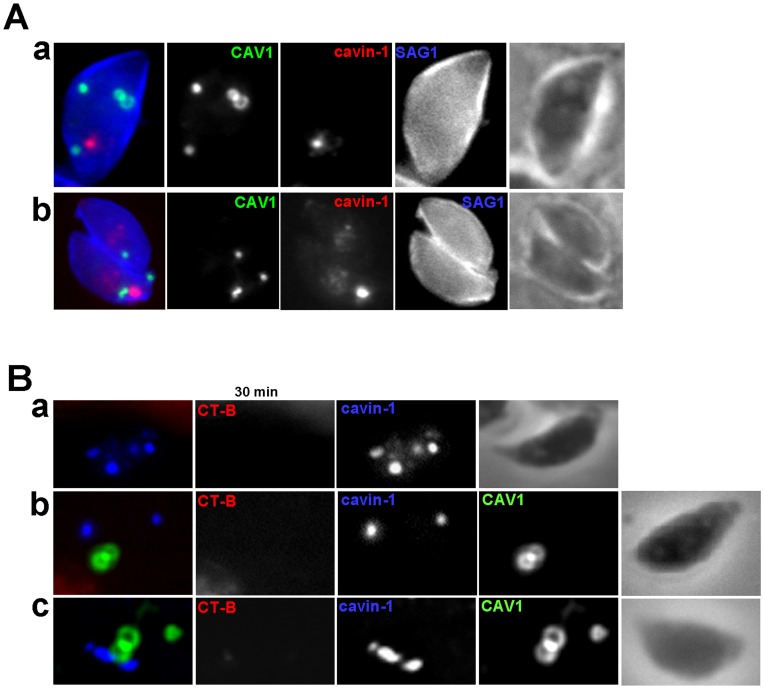
Assessment of caveolar endocytosis by CAV1-and/or cavin-1-expressing parasites. (A) IFA of *Toxoplasma* coexpressing CAV1-YFP cavin-1-HA immunolabeled with antibodies against HA (cavin-1-HA) and SAG1 (plasma membrane, blue) showing no overlap with the plasma membrane. (B) Assessment of Alexa Fluor 594- CT-B distribution after 30 min-pulse by fluorescence microscopy in cavin-1-HA-expressing parasites (panel a) and CAV1-YFP- and cavin-1-HA-expressing *Toxoplasm*a (panels b and c), showing no internalization of the CT-B regardless of the proximity of the structures containing CAV1 or cavin-1.

*Toxoplasma* expressing CAV1 and cavin-1 were immunolabeled with SAG1 to investigate a potential interaction of CAV1- and cavin-1-positive structures with the plasma membrane. We never observed any co-localization of CAV1 and cavin-1 with SAG1 at the plasma membrane of either non-dividing (Figure 13A, panel a) or replicating parasites (Figure 13, panel b). Nevertheless, we assayed these transgenic parasites for internalization of fluorescent CT-B for 30 min, compared to parasites expressing CAV1 (Figure 5) or cavin-1 (Figure 13B, panel a) alone. In line with the results obtained with single expressors, data from five independent experiments did not reveal any fluorescent CT-B staining of parasites expressing both CAV1 and cavin-1 regardless of the propinquity of CAV1-containing vesicles with cavin-1-positive structures (Figure13B, panels b and c). These data demonstrate the inability of transgenic parasites to internalize a caveolar ligand, and are in accordance with the absence of CAV1- and cavin-1-forming pits at the parasite’s plasma membrane.

## Discussion

In conclusion, expression of mammalian CAV1 in *Toxoplasma* results in the accumulation of CAV1-positive vesicles in the cytoplasm, without leading to the formation of morphologically defined caveolae inserted into the plasma membrane and the induction of caveolar endocytosis. Even the co-expression of CAV1 and cavin-1 does not reconstitute plasmalemmar caveolae for caveolae-dependent endocytosis. However, simultaneous expression of CAV1 and cavin-1 in *Toxoplasma* results in a dramatic entanglement of membranous structures containing CAV1 and cavin-1. This suggests that these proteins have a combined capacity to restructure membranes.

The CAV1-containing vesicles formed by transgenic *Toxoplasma* appear to correspond to mammalian exocytic caveolar precursor organelles en route to the plasma membrane based on the following observations: the detection of CAV1 on the parasite’s Golgi, CAV1 palmitoylation, the presence of CAV1 on vesicles in the post-Golgi region, and the topology of CAV1 at the periphery of vesicles. In mammalian cells, the coat of caveolins is very compact as it has been estimated that 144 CAV1 molecules are present per invagination [65]. By analogy to the mammalian system, the CAV1-containing vesicles in *Toxoplasma* show an abundant gold staining as observed by immunoEM using antibodies identifying CAV1, suggestive of a high density of CAV1 molecules at the vesicle’s periphery. *Toxoplasma* contains cholesterol and sphingolipid-rich lipid rafts [37], [38], which are two necessary lipidic components for driving the formation of caveolae vesicles in mammalian cells. It is plausible to envision that in transgenic parasites, CAV1 might also be packaged in detergent-resistant domains of the secretory pathway, leading to the biogenesis of typical pre-caveolae vesicles. Nevertheless, caveolae-induced vesicles in transgenic parasites are highly heterogeneous in diameter and vary from 30 to greater than 500 nm. In comparison, insect cells and bacteria transfected with *CAV1* form a homogeneous population of caveolae-sized vesicles with diameters between 50–120 nm [31] and 45–50 nm [32] respectively, which is closer to the size of caveolae in mammalian cells (∼50–100 nm). In this case, the heterogenous population of CAV1-induced vesicles in *Toxoplasma* may reflect the lack of structural factors that contribute to the canonical shape and size of caveolae of higher eukaryotic cells.

What could be the causes for the absence of caveolae pits at the surface of CAV1- and/or cavin-1-expressing *Toxoplasma*? A first possibility would be the lack of critical factors involved in the delivery of post-Golgi caveolae vesicles to the plasma membrane or in the construction of the caveolar neck in *Toxoplasma*. Supporting this idea, insect cells transfected with *CAV1* also accumulate caveolar vesicles in the cytoplasm [31]. In mammalian cells, the machinery for caveolae incorporation into the plasma membrane or scission includes cholesterol and the ganglioside GM_1_ at the cell surface, as well as dynamin, actin, and tyrosine kinases [4]. *Toxoplasma* expresses several homologues of these proteins, and therefore could have the competency to form endocytic vesicles. It has been shown that mammalian syntaxin 6 regulates the delivery of microdomain-associated GM_1_ and of proteins required for caveolar uptake to the cell surface [49]. Syntaxin 6 is a *t*-SNARE protein that localizes to the *trans*-Golgi network where it distributes and interacts with CAV1. To this end, the *Toxoplasma* genome also contains a putative SNARE domain-containing syntaxin 6 homolog (TGME49_100290 in www.ToxoDB.org), which may play a role analog to mammalian syntaxin 6. However, it remains possible that the fusion machinery of the parasite is different from that in mammalian cells, and not fully efficient to connect pre-caveolae vesicles with the plasma membrane. In mammalian cells, caveolin-2 is usually co-expressed with CAV1, and this protein regulates the number of plasma membrane attached caveolae [15]. Co-transfection of insect cells with *CAV1* and *caveolin-2* induces the formation of uniform caveolae-sized vesicles with a diameter of 45–65 nm, smaller in size than vesicles in CAV1-expressing insect cells [66]. However, the CAV1/caveolin-2-positive vesicles do not fuse with the plasma membrane, making it unlikely that the lack of caveolin proteins other than CAV1 is responsible for the incapability of *Toxoplasma* to form plasmalemmal caveolae upon CAV1and/or cavin-1 expression. In this case, it is more probable that other yet unidentified factors may play a critical role in the post-Golgi targeting of caveolar vesicles to the plasma membrane.

A second possibility for the absence of caveolar uptake in transgenic *Toxoplasma* would be due to the unique cytoarchitecture of the parasite imposed by the presence of the subplasmalemmar IMC. The parasite’s plasma membrane is tightly associated with the flattened membrane cisternae of the IMC [51]. Absent at the posterior end of *Toxoplasma*, the IMC runs along the length of the parasite with local interruptions for exocytic events. Its presence may then restrict the access of caveolae vesicles to the plasma membrane and interfere with the creation of caveolar invaginations at the surface, although no insertion of CAV1 into the plasma membrane at the basal extremity has been observed. Thirdly, the parasite’s plasma membrane may not be poised to form any invaginations to generate vesicles. In support of this idea is that, although clathrin molecules and adaptin effectors like the (AP)2 complex are expressed by *Toxoplasma*, the parasite never develops clathrin-coated pits at its surface nor internalizes cargos in a clathrin-dependent manner. More generally, no evidence exists that *Toxoplasma* can take up any macromolecules from the environment via an endocytic membrane system. This unique feature could be dictated by the intracellular lifestyle of the parasite living within a vacuole in mammalian cells where the acquisition of nutrients occurs by transport systems across the parasite’s plasma membrane [67].

While co-expression of mammalian CAV1 and cavin-1 does not reconstitute the caveolar endocytic pathway in *Toxoplasma*, it leads to a dramatic entanglement of membranous structures within the parasite. Solely in parasites co-expressing the two proteins, we observe a change in the morphology of the cavin-1-containing structures from vesicles to long membrane tubules, while CAV1-positive vesicles are more cylindrical in shape rather than spherical. In addition, we observe by transmission EM long and convoluted membrane whorls in the co-expressors. The molecular mechanism, by which CAV1 and cavin-1 collaborate to sculpt membranes in parasite co-expressors, remains to be clarified but evidently, the intertwining of CAV1- and cavin-1-containig structures, and partial co-localization of the two proteins on the same structure, suggest a coordinate interaction between CAV1 and cavin-1 to remodel membranes. Many studies favor this idea and have associated caveolar protein components with membrane dynamics. First, it is well known that caveolae function in lipid transport, membrane trafficking and vesicular transport [18]. CAV1 binds with high affinity to cholesterol and long chain unsaturated fatty acids, interacts with GM_1_, and moves these lipids within cells. Furthermore, caveolae bud from the plasma membrane of endothelial cells vesicles to form dynamic endocytic and transcytotic vesicles [68]. Second, CAV1 and cavin-1 drive the formation of caveolae, and a cooperation between these proteins is imperative for this process. In some cancer cells, the coordination between these proteins is lost, resulting in antagonistic effect between CAV1 and cavin-1. Expression of CAV1 in cancer cells stimulates the formation of long, branched tubules derived from the plasma membrane (up to 50 µm) [69]. In contrast, in cancer cells expressing both CAV1 and cavin-1, shorter tubules are visible, indicating that cavin-1 limits the ability of CAV1 to form tubules. While CAV1 promotes and cavin-1 inhibits membrane deformation in these cancer cells, conversely, the production of long membranous structures is exacerbated in CAV1- and cavin-1-expressing *Toxoplasma*. Regardless of the type of membrane deformations observed in the transfected cells, these findings strengthen the notion that a synergy between CAV1 and cavin-1 is important for proper cell functions, and that cavin-1 regulates CAV1 membrane dynamics. Third, besides forming caveolae, CAV1 is involved in the biogenesis of other membranous structures, such as invadopodia [70]. Invadopodia are formed *de novo* at the cell periphery. They represent ventral membrane protrusions involved in the degradation of the extracellular matrix. CAV1 assembles these protrusions by trafficking lipid raft components and matrix metalloproteinases at nascent invadopodia. This illustrates a significant role of CAV1 for organizing plasma membrane composition to create various specialized domains with well-defined functions. Forth, surface-located caveolae are membrane reservoir that can provide the additional membrane required to maintain membrane tension homeostasis. Upon mechanical stress, invaginated caveolae buffer membrane tension by flattening out in the plasma membrane [71]. The rapid disassembly of caveolae is triggered by the release of CAV1 and cavin-1 from invaginated caveolae at the plasma membrane. This defines a unique role of caveolae as membrane-mediated sensors and regulators of the plasma membrane tension.

Jointly, accumulating evidence support dynamic and coordinated roles of caveolin-1 and cavin-1 for regulating the physical and chemical composition of membranes, either to remodel existing membranes or build new membranous structures and organelles. The production of very long and intricately twisted membranes forming a whorl-like pattern by CAV1- and cavin-1-expressing *Toxoplasma* comforts this idea.

## Supporting Information

Figure S1 (A**)** Quantitative distribution of the number of *Toxoplasma* per PV either wild-type parasites (control, black histograms) or CAV1-expressing parasites (cav, grey histograms). Parasite development was monitored at the indicated time points p.i. for 2 independent infected monolayers. (B) Ultrastructure of CAV1-expressing parasites observed by EM 24 h p.i. showing normal PV with numerous dividing parasites (arrows). DG, dense granules; Go, Golgi; hcell, host cell; LB, lipid body; m, mitochondrion; rh, rhoptry.
(DOC)

Figure S2 Detection of GM_1_ species in *Toxoplasma*. (A–C) Chromatogram of a representative base methanolysed lipid extract of *T. gondi* generated by extraction of the ion at m/z 1518.8532, which corresponds to the GM1 species with a *N*-hexadecanoyl moiety. The experimental (MH)+ and (MNa)+ mass clusters of compound eluting at 5.36 in A are given in B. The exact and experimental mass clusters of the N-hexadecanoyl GM1 species are given in C, as well as assignment details.
(DOC)

Figure S3 Co-alignment of CAV1- and cavin-1-containing tubules. Fluorescence microscopy of transgenic parasites co-expressing CAV1-YFP and cavin-1-HA showing series of z-stacks of cavin-1- and CAV1-labeled vesicles in transgenic parasites (A) and panels of 3-D reconstruction of the series of z-stacks of showing rotated views of the image (B).
(DOC)
