# Supplementary material for: Introduction of Caveolae Structural Proteins into the Protozoan Toxoplasma Results in the Formation of Heterologous Caveolae but Not Caveolar Endocytosis
Source: PLoS One. 2012 Dec 14;7(12):e51773. doi: 10.1371/journal.pone.0051773 (PMC3522706; doi:10.1371/journal.pone.0051773)
Supplement: Figure S1 — (A) Quantitative distribution of the number of Toxoplasma per PV either wild-type parasites (control, black histograms) or CAV1-expressing parasites (cav, grey histograms). Parasite development was monitored at the indicated time points p.i. for 2 independent infected monolayers. (B) Ultrastructure of CAV1-expressing parasites observed by EM 24 h p.i. showing normal PV with numerous dividing parasites (arrows). DG, dense granules; Go, Golgi; hcell, host cell; LB, lipid body; m, mitochondrion; rh, rhoptry. (DOC) [file pone.0051773.s001.doc]

**A B**

**Figure S1**
