# Supplementary figures and images for: Introduction of Caveolae Structural Proteins into the Protozoan Toxoplasma Results in the Formation of Heterologous Caveolae but Not Caveolar Endocytosis
Source: PLoS One. 2012 Dec 14;7(12):e51773. doi: 10.1371/journal.pone.0051773 (PMC3522706; doi:10.1371/journal.pone.0051773)

**
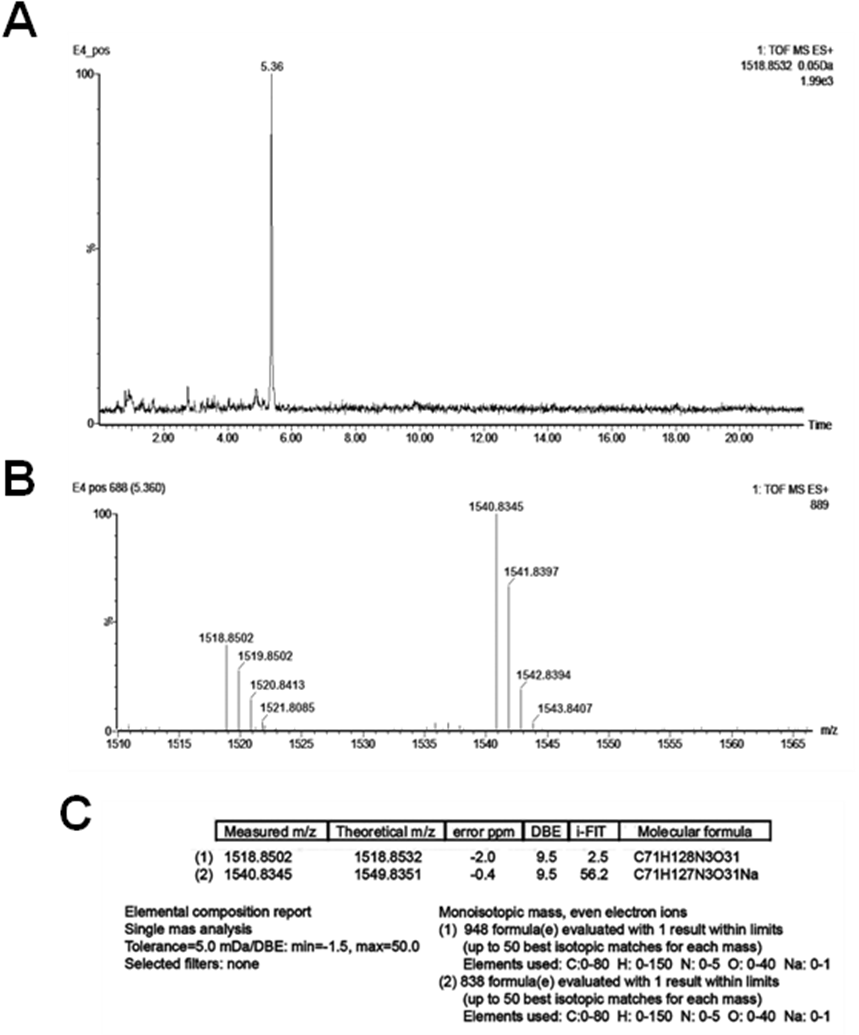
**

**Figure S2**

Supplement: Figure S2 — Detection of GM1 species in Toxoplasma. (A–C) Chromatogram of a representative base methanolysed lipid extract of T. gondi generated by extraction of the ion at m/z 1518.8532, which corresponds to the GM1 species with a N-hexadecanoyl moiety. The experimental (MH)+ and (MNa)+ mass clusters of compound eluting at 5.36 in A are given in B. The exact and experimental mass clusters of the N-hexadecanoyl GM1 species are given in C, as well as assignment details. (DOC) [file pone.0051773.s002.doc]
